# Plasma levels of soluble tumor necrosis factor-α receptors are related to total and LDL-cholesterol in lean, but not in obese subjects

**DOI:** 10.1186/1475-2840-5-14

**Published:** 2006-06-27

**Authors:** Marek Straczkowski, Irina Kowalska, Agnieszka Nikolajuk, Agnieszka Adamska, Malgorzata Karolczuk-Zarachowicz, Monika Karczewska-Kupczewska, Agnieszka Kozlowska, Maria Gorska

**Affiliations:** 1 Department of Endocrinology, Diabetology and Internal Medicine, Medical University of Bialystok, M.C. Sklodowskiej 24a, 15-276 Bialystok, Poland

## Abstract

**Background:**

Tumor necrosis factor-α (TNFα) is a mediator of insulin resistance. Plasma levels of soluble TNFα receptors (sTNFR1 and sTNFR2) probably reflect paracrine action of the cytokine. TNFα is also a regulator of lipid metabolism, however, data about impact of obesity on the relationships between TNFα and plasma lipids remain controversial.

**Aim:**

The purpose of the present study was to examine the associations of TNFα system with plasma lipids in lean and obese subjects with normal glucose metabolism.

**Methods:**

We examined 63 subjects, 33 lean (BMI<25 kg × m^-2^) and 30 with marked overweight or obesity (BMI>27.8 kg × m^-2^). Anthropometric and biochemical parameters were measured. Oral glucose tolerance test and euglycemic hyperinsulinemic clamp were also performed.

**Results:**

Obese subjects were markedly more insulin resistant and had higher levels of both TNFα receptors. Total (TC) and LDL-cholesterol (LDL-C), triglycerides (TG) and non-esterified fatty acids (NEFA) were also higher in the obese group. In obese subjects, both receptors were significantly related to TG and HDL-cholesterol (HDL-C), while sTNFR2 was also associated with NEFA. All those correlations disappeared after controlling for insulin sensitivity. In lean subjects, both receptors were related to TC, HDL-C and LDL-C. In that group, sTNFR1 predicted values of all those parameters independently of BMI, plasma glucose and insulin, and insulin sensitivity.

**Conclusion:**

We conclude that TNFα receptors are associated with plasma lipids in different way in lean and in obese subjects. TNFα system is probably important in determining cholesterol levels in lean subjects, while in obese this effect might be masked by other metabolic abnormalities.

## Background

Tumor necrosis factor-α (TNFα) is a cytokine involved in regulation of the whole-body carbohydrate and lipid metabolism. One of the main metabolic effects of TNFα action is the development of insulin resistance [1]. In insulin resistant states, the cytokine acts mostly in an auto- and paracrine manner in adipose tissue [2] and skeletal muscle [3]. Two cell surface TNFα receptors were described in humans, TNFR1 (p60) and TNFR2 (p80), soluble forms of those receptors (sTNFR1 and sTNFR2) are present in plasma and it is supposed that their concentrations, especially sTNFR2, might reflect local action of TNFα in tissues [4]. Soluble TNFR might neutralize TNFα at high levels, but they also might stabilize its bioactivity, help sequester TNFα to its membrane receptors and thus increase the effects of the cytokine [5]. Adipose tissue TNFR2 mRNA and protein and plasma levels of sTNFR2 are increased in obesity and related to insulin resistance [4,6], in those studies no differences in TNFR1 levels were observed. In contrast, other authors reported an increase in adipose tissue expression [7] and plasma levels [8] of both receptors in obese subjects. Plasma TNFα values are usually low and do not give the precise information about its auto- and paracrine action. It is propose that sTNFR2 might serve as the best predictor of local TNFα system activity [4].

There are data that TNFα might also be an important factor determining plasma cholesterol levels. The cytokine induces an increase in serum cholesterol and in hepatic hydro-3-methyl-glutaryl coenzyme A reductase activity in mice [9]. TNFα also induces maturation of sterol regulatory element binding protein-1 (SREBP-1), an important transcription factor in cholesterol biosynthesis [10]. There is an evidence that *TNFR2 *gene polymorphism is associated with hypercholesterolemia [11] and coronary artery disease [12]. However, data about plasma TNFα receptors as determinants of total and LDL-cholesterol levels and about impact of obesity on the relationships between TNFα system and plasma lipids remain controversial [13,14].

The aim of the present study was to examine the associations of TNFα system with plasma lipids in lean and obese subjects.

## Methods

### Subjects

A total of 63 subjects, 33 lean (BMI<25 kg × m^-2^, 14 men and 19 women) and 30 with marked overweight or obesity (BMI>27.8 kg × m^-2^, 12 men and 18 women) were recruited for the present study. The subjects were recruited actively from Outpatient Clinic of Department of Endocrinology, Diabetology and Internal Medicine of Medical University of Białystok. The basal characteristics of the studied groups are presented in Table 1. All the subjects were non-smokers, without ischaemic heart disease, hypertension, peripheral vascular disease, infections or any other serious medical problems. Only subjects without family history of type 2 diabetes were recruited for the present study. Before participating in the study, physical examination and resting electrocardiography were performed. All subjects underwent an oral glucose tolerance test (OGTT) and all had normal glucose tolerance according to WHO criteria. All subjects gave written informed consent before entering the study. The study protocol was approved by the Ethics Committee of Medical Academy, Białystok.

**Table 1:** Anthropometric parameters in the studied groups (mean ± SD).

	Lean subjects (n = 33)	Obese subjects (n = 30)
Age (year)	35.24 ± 7.56	37.07 ± 9.68
BMI (kg × m^-2^)	22.23 ± 2.14	32.21 ± 3.99*
WHR	0.80 ± 0.06	0.88 ± 0.08*
Percent of body fat	17.00 ± 5.16	36.48 ± 9.36*

BMI, body mass index; WHR, waist-to-hip ratio.

*p < 0.05 in obese vs lean subjects.

### Anthropometry

All analyses were performed after an overnight fast. The BMI was calculated as body weight × height ^-2 ^(kg/m^2^). The waist-to hip ratio (WHR) was also estimated. The waist circumference was measured at the smallest circumference between the rib cage and the iliac crest, with the subject in the standing position. The hip circumference was measured at the widest circumference between the waist and the thighs. Percent of body fat was estimated by bioelectric impedance analysis using the Tanita TBF-511 Body Fat Analyzer (Tanita Corp., Tokyo, Japan), fat mass (FM) and fat-free mass (FFM) were calculated.

### Insulin sensitivity

Insulin sensitivity was evaluated by the euglycemic hyperinsulinemic clamp technique according to DeFronzo et al [15], as described previously [16,17]. On the morning of the study, two venous catheters were inserted into antecubital veins, one for the infusion of insulin and glucose and the other in the contralateral hand for blood sampling, that hand was heated to approximately 60°C. Insulin (Actrapid HM, Novo Nordisk, Copenhagen, Denmark) was given as a primed-continuous intravenous infusion for 2 hours at 40 mU × m^-2 ^× min^-1^, resulting in constant hyperinsulinemia of approximately 550 pmol/l. Arterialized blood glucose was obtained every 5 minutes and 20% dextrose (1.11 mol/l) infusion was adjusted to maintain plasma glucose levels at 5.0 mmol/l. The glucose infusion rate approached stable values during final 40 minutes of the study and the rate of whole-body glucose uptake (M value) was calculated as the mean glucose infusion rate from 80 to 120 min, corrected for glucose space and normalized per kilogram of fat-free mass (M/FFM).

### Other analyses

Fasting blood samples were also taken from the antecubital vein for the determination of glycated hemoglobin (HbA1c), plasma lipids, TNFα, sTNFR1 and sTNFR2. For the determination of plasma TNF system samples were frozen at -70°C.

### Analytical procedures

Plasma glucose was measured immediately by the enzymatic method using glucose analyzer (YSI 2300 STAT Plus, Yellow Spring Instuments, OH). Plasma insulin was measured with the Medgenix Enzyme Amplified Sensitivity Immunosorbent Assay (EASIA) test (BioSource Europe, Nivelles, Belgium). The minimum detectable concentration was 1.05 pg/l and the intra-assay and inter-assay coefficients of variation (CVs) were below 5.5% and 10%, respectively. In that method, human and animal proinsulins present no cross-reaction. HbA1c were measured by the high-performance liquid chromatography method (Bio-Rad, Muenchen, Germany). Plasma total (TC) and HDL-cholesterol (HDL-C) and triglycerides (TG) were assessed by the enzymatic methods (Cormay, Warsaw, Poland). Plasma LDL-cholesterol (LDL-C) was calculated from the Friedewald's formula. Plasma non-esterified fatty acids (NEFA) were measured by colorimetric method [18].

Plasma TNFα concentrations were measured by the Immunoassay Kit (BioSource International, Camarillo, CA, USA) with the minimum detectable concentration 1.7 pg/ml and with the intra-assay and inter-assay CVs below 5.2% and 8.5%, respectively. Plasma sTNFR1 and sTNFR2 were determined with the EASIA kits (BioSource Europe). The minimum detectable concentration was 0.05 ng/ml for sTNFR1 and 0.1 ng/ml for sTNFR2. The intra-assay and inter-assay CVs for both receptors were below 6.5% and 9%, respectively. sTNFR1 EASIA does not cross react with sTNFR2 and TNFα does not interfere with the assay.

### Statistical analysis

The statistics were performed with the STATISTICA 5.0 program (StatSoft, Krakow, Poland). Differences between the groups were evaluated with an unpaired Student's t-test. Relationships between variables were estimated with Pearson correlation coefficient analysis and with multiple regression analysis. Variables, which did not have normal distribution (insulin, TG) were log-transformed before analyses. The level of significance was accepted at p value less than 0.05.

## Results

Biochemical parameters of the studied groups are presented in Table 2. Obese subjects had higher levels of HbA1c, plasma glucose (both p < 0.001) and insulin (p < 0.005). The obese group was also markedly more insulin resistant (p < 0.005) and had higher levels of sTNFR1 (p < 0.0005) and sTNFR2 (p < 0.02). Plasma TNFα did not differ between the studied groups.

**Table 2:** Biochemical parameters in the studied groups (mean ± SD).

	Lean subjects (n = 33)	Obese subjects (n = 30)
HbA1c (%)	5.34 ± 0.41	5.78 ± 0.50*
Plasma glucose (mmol/l)	4.86 ± 0.48	5.35 ± 0.57*
Plasma insulin (pmol/l)	65.84 ± 32.14	105.33 ± 68.60*
M/FFM (μmol × kg^-1 ^× min^-1^)	50.50 ± 14.64	39.37 ± 12.44*
Plasma TC (mmol/l)	4.43 ± 0.78	5.52 ± 1.08*
Plasma TG (mmol/l)	1.02 ± 0.52	1.64 ± 0.93*
Plasma HDL-C (mmol/l)	1.37 ± 0.32	1.23 ± 0.41
Plasma LDL-C (mmol/l)	2.59 ± 0.81	3.52 ± 0.96*
Plasma NEFA (mmol/l)	0.347 ± 0.116	0.504 ± 0.155*
Plasma TNFα (pg/ml)	4.89 ± 1.53	5.22 ± 2.69
Plasma sTNFR1 (ng/ml)	1.94 ± 0.37	2.36 ± 0.44*
Plasma sTNFR2 (ng/ml)	4.12 ± 0.79	4.74 ± 1.20*

HbA1c, glycated hemoglobin; M/FFM, whole-body glucose uptake normalized per kg of fat-free mass; TC, total cholesterol; TG, triglycerides; HDL-C, HDL-cholesterol; LDL-C, LDL-cholesterol; NEFA, non-esterified fatty acids; TNFα, tumor necrosis factor α; sTNFR1, soluble TNFα receptor 1; sTNFR2, soluble TNFα receptor 2.

*p < 0.05 in obese vs lean subjects.

Plasma TC, LDL-C (both p < 0.0001), TG (p < 0.005) and NEFA (p < 0.00005) were also higher in the obese group. No difference in HDL-C levels was observed between the studied groups. When we analysed all the studied population, we observed significant correlations of both sTNFR1 and sTNFR2 with TC (r = 0.38, p < 0.005 and r = 0.31, p < 0.02), TG (r = 0.49, p < 0.0005 and r = 0.42, p < 0.001), HDL-C (r = -0.49, p < 0.0005 and r = -0.39, p < 0.005), LDL-C (r = 0.41, p < 0.001 and r = 0.32, p < 0.02) and NEFA (r = 0.48, p < 0.0005 and r = 0.40, p < 0.001, respectively). The correlations between TNFα system and plasma lipids were different among groups when lean and obese subjects were analysed separately.

**Figure 1:**
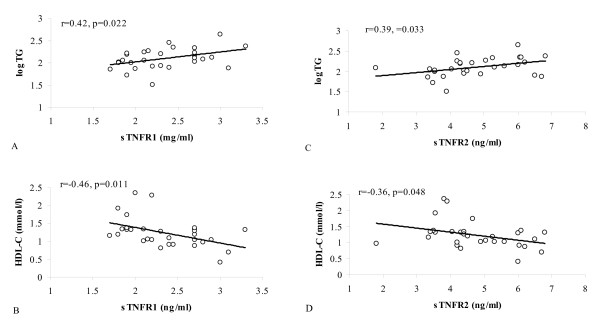
Relationships between soluble TNFα receptors and plasma lipids in obese subjects. Relationships of: A. sTNFR1 and TG, B. sTNFR1 and HDL-C, C. sTNFR2 and TG, D. sTNFR2 and HDL-C.

In obese subjects, both receptors were significantly related to TG and HDL-C (Fig. 1), while sTNFR2 was also associated with NEFA (r = 0.43, p < 0.05). All those correlations disappeared after controlling for insulin sensitivity.

**Figure 2:**
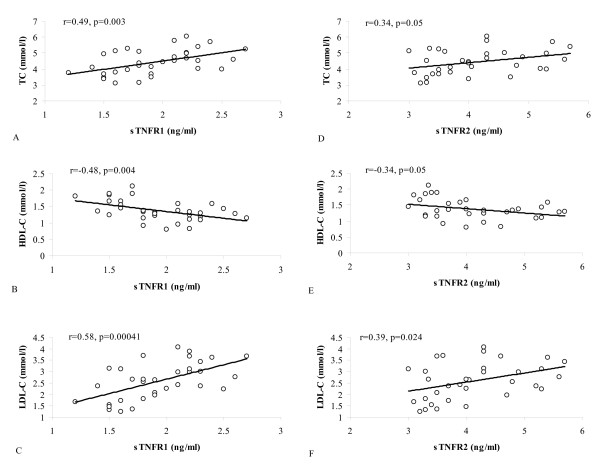
Relationships between soluble TNFα receptors and plasma lipids in lean subjects. Relationships of : A. sTNFR1 and TC, B. sTNFR1 and HDL-C, C. sTNFR1 and LDL-C, D. sTNFR2 and TC, E. sTNFR2 and HDL-C, F. sTNFR2 and LDL-C.

In lean subjects, both receptors were related to TC, HDL-C and LDL-C (Fig. 2). All the relationships were stronger for sTNFR1 than for sTNFR2. The correlations with TG and NEFA did not reach the level of significance. Multiple regression analysis revealed that sTNFR1, but not sTNFR2, predicted plasma levels of TC (R^2 ^= 0.24, p < 0.005), HDL-C (R^2 ^= 0.23, p < 0.005) and LDL-C (R^2 ^= 0.33, p < 0.0005) independently of BMI, plasma glucose, insulin, and insulin sensitivity.

## Discussion

We demonstrated an increase in both sTNFR1 and sTNFR2 in the obese group. This is in agreement with our previous results [19], although in a smaller study we were able to detect an increase only in sTNFR2 [20]. The present study shows that soluble forms of TNFα receptors are related to plasma lipids in different way in lean and in obese subjects.

In the obese, both receptors were associated positively with TG and negatively with HDL-C, thus contributing to lipoprotein profile typical for the insulin resistance syndrome. The relationships between TNFα receptors and plasma lipids in the obese group disappeared after controlling for insulin sensitivity, suggesting that lipid abnormalities associated with TNFα system in obesity might be fully explained by TNFα-associated insulin resistance. In contrast, in the lean group sTNFR1 and sTNFR2 were related to TC, HDL-C and LDL-C, relationships of sTNFR1 were independent of insulin sensitivity and other examined parameters.

The present study does not show any cause-effect relationships. It was reported that accumulation of cholesteryl esters in macrophages exposed to LDL-immune complexes is related to increase in TNFα synthesis and release [21]. Hypercholesterolemic rabbits [22] and LDL-receptor knockout mice [23] present increased TNFα secretion. However, in a situation when an increase in TNFα or its receptors might be secondary to hypercholesterolemia, one may expect a significant relationships between TNFα system and cholesterol rather in the obese group, presenting higher lipid levels and higher risk of accelerated atherogenesis. Therefore, it seems unlikely that the above reports might help explaining findings of the present study. It is more probable that soluble TNFRs might reflect TNFα influence on cholesterol metabolism. The possible mechanism might be associated with an increase in hepatic hydro-3-methyl-glutaryl coenzyme A reductase activity [9] and/or maturation of SREBP-1 [10].

In recent studies, a microsatellite marker with 5 alleles in intron 4 of *TNFR2 *gene was reported [24]. This locus was associated with familial combined hyperlipidemia [24], hypertension, hypercholesterolemia [11] and coronary artery disease [12]. The genotypic effect on plasma sTNFR2 levels was also found [11,12]. It is suggested, that the effects of TNFα on lipid metabolism are influenced by *TNFR2 *genotype [11]. No data about *TNFR1 *gene polymorphism and lipid abnormalities are available.

Data about plasma TNFα receptors as determinants of total and LDL-cholesterol levels and about impact of obesity on the relationships between TNFα system and plasma lipids remain controversial. Both sTNFR1 and sTNFR2 were related to TC, TG and LDL-C in myotonic dystrophy patients [25]. It was also observed that both receptors were independent determinants of TC and LDL-C in healthy subjects, when lean and obese subjects were analysed together [13]. Plasma sTNFR2, but not sTNFR1, was associated with HDL2-cholesterol. In a large study conducted on 268 men in a wide range of BMI, both sTNFRs were related to TC and HDL-C [14]. In that study, however, TNFα receptors were not independent predictors of plasma lipids and, in contrast to insulin, did not significantly change an association between BMI and cardiovascular risk factors [14].

Our data show, that TNFα is especially important in determining plasma total cholesterol and its fractions in lean subjects. This effect is probably independent of insulin sensitivity. We observed that lean normoglycemic insulin-resistant offspring of type 2 diabetic subjects had higher plasma levels of sTNFR2 [26], and these levels are associated with lower plasma adiponectin in that group [27]. When we analysed offspring and control group together, we observed similar correlations to those reported here. To exclude the effect of diabetes-prone genotype, only subjects with no family history of type 2 diabetes were recruited for the present study. Our findings provide further evidence that TNFα system might be involved in the pathogenesis of metabolic syndrome even before the onset of obesity and indicate that its metabolic actions may extend beyond inducing insulin resistance.

In obese subjects probably there are other factors, genetic or environmental (for instance nutrition), associated with the accumulation of body fat, that are more important in determining TC and its fractions than TNFα itself. It is likely that those factors could mask the TNFα effect on cholesterol metabolism. Insulin resistance, which is associated with TNFα overactivity in obesity, might also influence lipid metabolism. Alternatively, TNFα may induce accelerated atherogenesis in obesity by other mechanisms. We demonstrated that plasma sTNFR2 increase in parallel with soluble intercellular adhesion molecule-1 (sICAM-1) [28] and interleukin 8 [29] in obese subjects. In the study of Elkind et al [30], both receptors predicted maximal carotid plaque thickness independently of LDL-C and other parameters, like BMI, diabetes or hypertension.

## Conclusion

We conclude that TNFα receptors are associated with plasma lipids in different way in lean and in obese subjects. TNFα system is probably important in determining cholesterol levels in lean subjects, while in obese this effect might be mediated by other metabolic abnormalities.

## Abbreviations

Tumor necrosis factor α (TNFα)

Soluble tumor necrosis α receptor 1 (sTNFR1)

Soluble tumor necrosis α receptor 2 (sTNFR2)

Body mass index (BMI)

Total cholesterol (TC)

LDL – cholesterol (LDL-C)

Triglycerydes (TG)

Non – esterified fatty acids (NEFA)

HDL – cholesterol (HDL – C)

Sterol regulatory element binding protein 1 (SREBP)

Oral glucose tolerance test (OGTT)

Waist – to hip ratio (WHR)

Fat mass (FM)

Fat – free mass (FFM)

Hemoglobin A1c (HbA1c)

Soluble intercellular adhesion molecule – 1 (sICAM – 1)

Intra-assay and inter-assay coefficients of variation (CVs)

## Competing interests

The author(s) declare that they have no competing interests.

## Authors' contributions

Marek Straczkowski and Irina Kowalska conceived and designed the study as well as statistical analysis and wrote the manuscript; Agnieszka Nikolajuk participated in the clamp studies and performed immunoassays; Agnieszka Adamska, Monika Karczewska – Kupczewska, Malgorzata Karolczuk-Zarachowicz, Agnieszka Kozlowska participated in clinical part of the studies as well as in a clamp studies; Maria Gorska participated in design and coordination of the study.

All authors read and approved the final manuscript.
